# Intraoperative Methadone Versus Non-Methadone Analgesia in Pediatric Cardiac Surgery: A Retrospective Cohort Study

**DOI:** 10.3390/children12050567

**Published:** 2025-04-28

**Authors:** Brian Blasiole, Danielle R. Lavage, Hsing-Hua Sylvia Lin, Scott E. Licata, Sahana Sivam, Inesh Sivam, Laura M. Le, Senthilkumar Sadhasivam

**Affiliations:** 1 Department of Anesthesiology and Perioperative Medicine, School of Medicine, University of Pittsburgh, Pittsburgh, PA 15261, USA; blasioleb@upmc.edu (B.B.); lavagedr@upmc.edu (D.R.L.); hsl26@pitt.edu (H.-H.S.L.); licatase@chp.edu (S.E.L.); 2 UPMC Children’s Hospital of Pittsburgh, Pittsburgh, PA 15224, USA; 3 North Allegheny High School, Pittsburgh, PA 15090, USA; ssivam@nastudents.org (S.S.); isivam@nastudents.org (I.S.); 4 School of Medicine, University of Pittsburgh, Pittsburgh, PA 15213, USA; lml89@pitt.edu

**Keywords:** children, cardiac surgery, methadone, pain scores, opioid use, postoperative nausea and vomiting

## Abstract

Introduction: Methadone is an opioid-sparing opioid and it is increasingly used in children undergoing surgery due to its beneficial effects on postoperative pain scores, decreased opioid requirements, and fewer adverse effects compared to other opioids. Intraoperative methadone is not well studied in pediatric cardiac surgery. We hypothesized that intraoperative methadone-based analgesia would provide comparable effectiveness in pain management to non-methadone-based analgesia, including caudal morphine, following pediatric cardiac surgery. Methods: We conducted a retrospective cohort study of 287 children undergoing cardiac surgery using single institutional electronic health records with Society of Thoracic Surgeons database outcomes. Patients were administered intravenous opioids plus caudal morphine (≤6 years) or intravenous opioids in the non-methadone group versus intravenous methadone (two 0.1 mg/kg doses given intraoperatively) with or without additional intraoperative opioids. The primary outcome was postoperative opioid use in morphine milligram equivalents (MME)/kg. Results: This study included 287 pediatric cardiac surgical patients with a mean age of 3.8 years, 59% male, and 72% White. Among 287 patients, 67 (23%) received intraoperative methadone. Unadjusted analysis showed the methadone group had lower postoperative opioid use on the day of surgery (median = 0.3 vs. 0.5 MME/kg, *p* = 0.005). Adjusted analyses showed there were no significant differences in postoperative opioid use, average pain, maximum pain, antiemetic use, reintubation, and use of naloxone between methadone and non-methadone groups. Hospital length of stay was 2.62 times longer (95% CI: [1.55, 4.41] *p* < 0.001) in the methadone group vs non-methadone group, but this was only shown in the younger children (≤6 years), who also had higher max pain scores in the methadone group. All outcomes were similar between analgesia groups in older children (>6 years). Conclusions: Intraoperative methadone-based analgesia had comparable effectiveness in postoperative opioid use, pain, and antiemetic use compared to non-methadone-based intraoperative pain management for pediatric cardiac surgery. Large prospective studies of perioperative methadone are needed to examine methadone’s analgesic benefits in children undergoing cardiac surgery.

## 1. Introduction

Analgesic approaches for pediatric patients undergoing cardiac surgery demand consideration of the painful sternotomy and chest tube placement, a lengthy procedure with possible significant blood volume changes, consideration for fast-tracking and extubation in the operating room, and post-operative sedation. Short-acting narcotics such as fentanyl with or without neuraxial morphine have been the mainstay of analgesic approaches in these patients. Despite fentanyl’s rapid pain relief, its use can lead to respiratory depression, bradycardia, nausea, vomiting, and opioid tolerance [1,2]. Fentanyl’s short half-life demands frequent bolus doses, resulting in variable opioid levels, while prolonged infusions result in undesirable long context-sensitive half-life [3]. Prolonged opioid use during these cardiac surgical procedures can lead to increased tolerance and longer hospital stays, particularly in patients with previous opioid exposure [4].

Neuraxial techniques have long been utilized as a supplemental method to decrease opioid use in pediatric cardiac surgery. However, their application is restricted due to concerns about spinal or epidural hematoma formation when patients undergo full heparinization for cardiopulmonary bypass [5,6,7].

Methadone is a long-acting opioid and NMDA antagonist and is considered an opioid-sparing opioid. The intraoperative and early post-operative use of methadone has increased because of its demonstrated benefits, including fewer adverse effects like nausea and vomiting, reduced postoperative pain scores, and decreased pro re nata opioid use compared to alternative opioids [8,9]. Similarly, these benefits have led to the gain in popularity of methadone use in children undergoing surgery [10,11]. Early administration of methadone could potentially reduce the need for opioids and sedatives during surgery, simplifying medication management and potentially decreasing complications like delayed extubation and increased risk of apnea.

However, there exists limited data on the use of intraoperative methadone in children undergoing cardiac surgery [12]. In this large retrospective study, we investigated how administration of intraoperative methadone compared with non-methadone analgesia in children undergoing cardiac surgery.

We hypothesized that intraoperative intravenous methadone-based analgesia would provide comparable effectiveness in pain management to non-methadone-based opioid analgesia, including neuraxial (caudal) morphine, after pediatric cardiac surgery. Our hypothesis was formulated to evaluate whether intraoperative methadone provides comparable analgesic effectiveness to standard non-methadone opioid regimens, including caudal morphine, rather than aiming to demonstrate superiority. This noninferiority framework was chosen intentionally, as our primary interest was in assessing whether intraoperative methadone could offer similar clinical outcomes while potentially streamlining care (e.g., avoidance of neuraxial techniques).

## 2. Methods

### 2.1. Study Population

This retrospective cohort analysis utilized validated institutional electronic health record (EHR) data and data from the Society of Thoracic Surgeons (STS) Pediatric Cardiac Surgery Database. This study was approved by the University of Pittsburgh Institutional Review Board (STUDY20050148). We conducted a single-center review of pediatric patients less than 18 years old undergoing cardiac surgery between August 2021 and August 2023 at the UPMC Children’s Hospital of Pittsburgh. Patients included in the analysis were outpatients prior to surgery and were extubated in the operating room after the surgery. We excluded any patients who were inpatient or receiving narcotic medications for pain or sedation at least one month before surgery. We also excluded patients who were to undergo emergency surgery, cardiothoracic transplantation, or those requiring post-operative mechanical circulatory support.

### 2.2. Anesthetic Management

Patients in the non-methadone group were administered narcotics such as fentanyl or sufentanil. In general, non-methadone patients under 6 years old were administered a single-shot caudal epidural nerve block after general anesthesia induction consisting of bupivacaine 0.25% (1 mL-kg^−1^ up to 20 mL), preservative-free morphine (40 mcg-kg^−1^ up to 2.5 mg), and clonidine (2 mcg-kg^−1^). These patients also received a dose of intravenous fentanyl (1 mcg-kg^−1^) prior to incision. A second dose of fentanyl (1 mcg-kg^−1^) was administered upon initiation of cardiopulmonary bypass (CPB) when CPB was indicated. Additional fentanyl was administered before emergence at the discretion of the anesthesiologist. Analgesic approaches for non-methadone patients who did not receive a caudal morphine consisted of either fentanyl boluses, fentanyl infusion of 1–2 mcg-kg^−1^ per hour, or sufentanil infusion of 0.2–0.3 mcg-kg^−1^ per hour. Patients in the methadone group were administered methadone (0.1 mg-kg^−1^ up to 10 mg) on induction of anesthesia. A second dose of methadone (0.1 mg-kg^−1^ up to 10 mg) was administered on initiation of CPB when CPB was used. Patients in the methadone group were administered additional fentanyl boluses on emergence of anesthesia at the discretion of the anesthesiologist. Postoperative analgesia was standardized for ICU and acute care floors.

### 2.3. Outcome Measurements

Primary outcome: Daily postoperative opioid use in morphine milligram equivalent (MME)/kg was calculated based on established methods [13,14]. Secondary outcomes: (1) Average and max pain scores were based on the Face, Legs, Activity, Cry, Consolability (FLACC) pain scales [15,16]; (2) postoperative antiemetics was defined as receiving ondansetron during POD 0–2 for postoperative nausea and vomiting; (3) hospital and ICU length of stay; (4) need for reintubation; and (5) use of naloxone.

### 2.4. Statistical Analysis

Descriptive statistics are reported with means and standard deviations describing continuous distributions and counts and percentages describing categorical distributions. Non-normal continuous distributions were described with medians and interquartile ranges. Between treatment group differences were tested on continuous distributions using *t*-tests and Mann–Whitney U tests. Chi-squared tests and Fisher’s exact tests were applied to test for categorical differences. In EMR-based retrospective studies, treatment assignment is not randomized. To approximate a randomized controlled trial, inverse probability weighting (IPW) was used to balance observed confounding variables between the methadone and non-methadone groups. IPW was derived from propensity scores, which were estimated by fitting a logistic regression model with the analgesia treatment groups as the outcome and age, sex, and race, The Society of Thoracic Surgeons—European Association for Cardio-Thoracic Surgery (STAT) Mortality Categories, and intraoperative MME/kg as confounders.

IPW distributions were truncated at the 99th percentile. Diagnostics of histograms and measures of spread were analyzed to ensure common support of IPW between treatment groups. Standardized mean differences (SMDs) for demographics between treatment groups were reviewed before and after IPW weighting, with SMDs < 0.2 indicating them being balanced. Supplemental Table S1 presents the confounding variables included in the propensity score model and their corresponding SMDs pre- and post-IPW. Age and STAT category had SMDs > 0.2 before weighting, which were reduced to <0.2 after weighting. Outcome summary statistics were calculated using unadjusted data and IPW-adjusted pseudo population data. Longitudinal linear IPW adjusted mixed models were fitted to analyze between methadone-group comparisons over time for primary outcomes of MME/kg and pain scores. Random effects were fitted for each patient to adjust for within person variance. Fixed effects were fitted for both time and treatment group. Time points modeled were POD 0–2. Interaction terms between time and treatment group were analyzed. Interaction plots were visualized to understand interaction model estimates.

Secondary outcomes of postoperative nausea and vomiting (PONV, as measured by the use of postoperative antiemetics) were modeled using log-binomial IPW regression. Postoperative LOS was modeled using log gamma IPW regression models.

To address selection bias of age-based anesthesia protocol usage, all analyses were then replicated and stratified by ages six and under or greater than six. SMD imbalance and significant *p*-value were found for intraoperative MME/kg in the older than 6 cohort and so were included in both the IPW and a covariate in the outcome models. Due to limited sample size, interaction terms were not tested.

IPW adjusted analyses were performed using complete case analysis. Data management and statistical analysis were conducted in R software (version 4.3.1, R Core Team). The lmerTest package (version 3.1-3) was used to fit IPW mixed models, the survey package (version 4.4-2) was used to fit IPW adjusted summary stats and generalized linear models, and WeightIt (version 1.4.0) was used to calculate propensity scores and IPW. *p*-values < 0.05 was chosen as our decision rule for statistical significance.

## 3. Results

### 3.1. Baseline Characteristics and Analgesia Groups

The overall analytic cohort included 287 patients with a mean age of 3.8 years, 59% were male, 72% were White, 94% received CPB cardiovascular surgery, and 58% with STAT category one. Among 287 patients, 220 (77%) did not receive intraoperative methadone and 67 (23%) received intraoperative methadone. Sex, race, and STAT category were similar between the two study groups (Table 1). Patients were significantly older in the methadone group compared to non-methadone group (mean 7.6 vs. 2.7 years, *p* < 0.001) (Table 1) because caudal morphine with intravenous fentanyl (i.e., 78% in the non-methadone group, Table 2) was commonly used for children under 6 of age per standardized enhanced recovery after pediatric cardiac surgery. A total of 30/226 (13%) in ≤6 years old and 37/61 (61%) in >6 years received intraoperative methadone (Table 1). Intraoperative opioid was similar between methadone vs non-methadone group (median = 0.5 in MME/kg for both groups, *p* = 0.65, Table 2) in the overall sample.

### 3.2. Primary Outcome—Postoperative Opioid Consumption (MME/kg)

**Figure 1:**
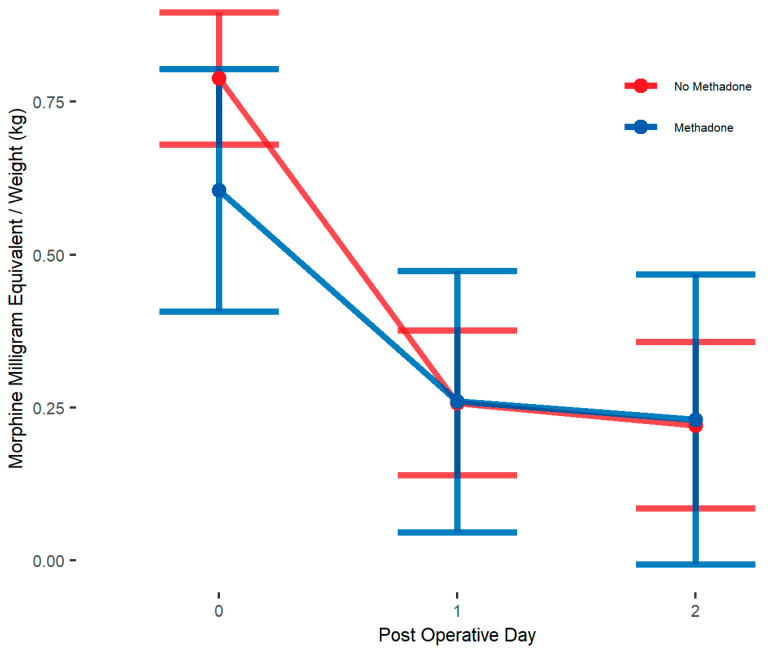
Unadjusted linear mixed model interaction plot showing MME/kg for whole sample cohort on PODs 0–2, by methadone group. Estimates showing model estimated means and confidence intervals around them. Interaction terms are included in estimates. *p*-values of group differences in each POD are POD 0 = 0.112, POD 1 = 0.986, POD 2 = 0.948. We observe no significant between-group differences by day.

**Figure 2:**
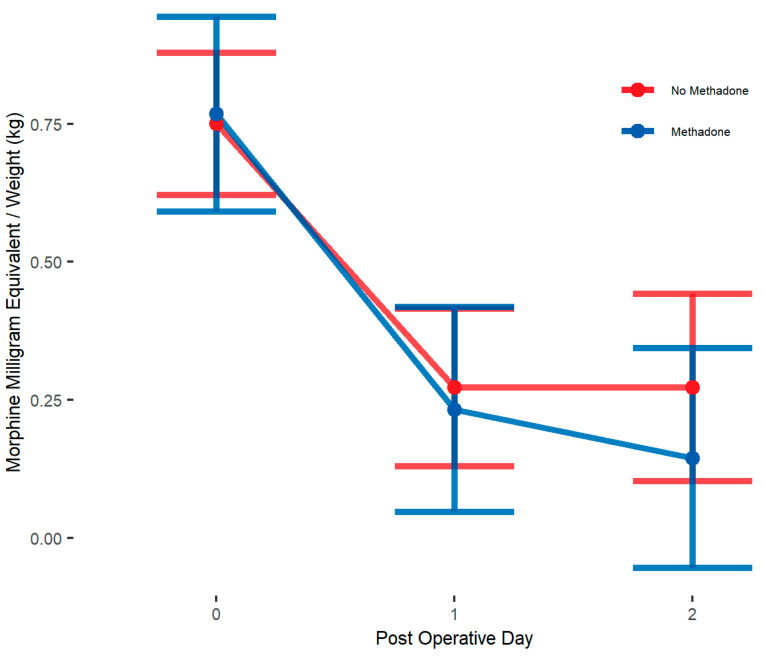
IPW adjusted linear mixed model interaction plot showing MME/kg for whole sample cohort on PODs 0–2, by methadone group. Estimates showing model estimated means and confidence intervals around them. Interaction terms are included in estimates. *p*-values of group differences in each POD are POD 0 = 0.874, POD 1 = 0.735, POD 2 = 0.338. We observe no significant between-group differences by day.

Unadjusted analysis showed that the primary outcome, postoperative opioid in MME/kg, was significantly lower in the methadone group compared to the non-methadone group (median = 0.3 vs. 0.5; *p* = 0.005) on POD 0. Opioid use on POD 1 and 2 was similar between treatment groups (Table 3). IPW adjusted medians showed opioid use to be not significantly different on POD 0, 1, and 2 (Table 4). IPW adjusted linear mixed models showed patients in methadone group had similar postoperative opioid use (β = −0.03, 95% CI: [−0.22, 0.16] MME/kg; *p* = 0.73, Table 5) compared to those in non-methadone group over POD 0–2 and there was no significant interaction between postoperative days and methadone group (Figure 1). Age stratified analysis using IPW adjusted linear mixed models shows no differences in opioid use across POD 0–2 between methadone analgesia groups for either age group (Figure 2) (Table 5).

### 3.3. Secondary Outcomes

Postoperative Pain. Unadjusted and IPW adjusted means showed no differences in average pain and max pain scores between groups (Table 3 and Table 4). IPW adjusted linear mixed models showed similar average pain scores (β = −0.07, 95% CI: [−0.4, 0.26]; *p* = 0.66) and max pain scores (β = −0.002, 95% CI: [−0.76, 0.76]; *p* = 0.99) between treatment groups (Table 5). Average pain was not significantly different in the age ≤6 group (β = 0.03, 95% CI: [−0.37, 0.44]; *p* = 0.87); however, max pain was significantly higher in the methadone group among children ≤6 years (β = 0.91, 95% CI: [0.15, 1.68]; *p* = 0.02). In children aged >6 years, no group differences were found in either max or average pain across PODs (Table 5).

Postoperative Antiemetics. Unadjusted analysis showed 40% in the methadone group receiving any postoperative antiemetics over POD 0–2 compared to 15% in the non-methadone group (*p* < 0.001, Table 3). Adjusted analysis showed 20.8% in the methadone group receiving any postoperative antiemetics over POD 0–2 compared to 17.5% in the non-methadone group (*p* = 0.56, Table 4). Mixed models also showed no differences for antiemetic use over POD 0–2 (relative risk = 1.19, 95% CI: [0.67, 2.1]; *p* = 0.56, Table 4) in the overall sample and no significant differences in either age group.

Length of Stay. Unadjusted analysis showed no differences in hospital LOS or ICU LOS between methadone groups (Table 3). Younger children in the methadone group (n = 30) had a longer median LOS compared to those in the non-methadone group (n = 196) (8 vs. 5 days, *p* = 0.012). In contrast, no difference was observed among older children (median LOS 4 vs. 4 days, *p* = 0.056). IPW adjusted median showed significantly higher LOS in the methadone group vs non-methadone group (median 8 vs. 4 days, *p* = 0.002, Table 4) and mean of LOS was 2.62 times longer in the methadone group (95% CI: 1.55, 4.41; *p* < 0.001, Table 5) than the non-methadone group in the overall sample. The IPW-adjusted analysis stratified by age confirmed that this relationship was particularly shown in the age ≤6 years group (mean ratio = 2.14, 95% CI: 1.3, 3.55; *p* < 0.001) but not in the age >6 years cohort (mean ratio = 1.13, 95% CI: 0.87, 1.46; *p* = 0.36, Table 5).

Additionally, there were no differences in the need for reintubation (13.3% vs. 9.7%, *p* = 0.52) and use of postoperative naloxone (0.7% vs. 2.9%, *p* = 0.17) between the methadone and non-methadone analgesia groups (Table 4).

## 4. Discussion

This is the first large, single-center, retrospective study in pediatric cardiac surgery patients demonstrating that intraoperative intravenous methadone provides comparable analgesia to non-methadone analgesic approaches. One notable observed finding was the reduction in immediate postoperative opioid requirements on the day of surgery with intraoperative methadone use in the unadjusted analysis. However, this difference was no longer statistically significant after IPW weighting, suggesting that multiple confounding variables influence postoperative opioid consumption. In addition, IPW adjusted analyses showed there were no differences in postoperative opioid use, average pain, max pain, antiemetic use, reintubation, and use of naloxone between methadone and non-methadone groups.

In this study, children who received intraoperative methadone were generally older (>6 years). Hospital length of stay was longer in the methadone group versus the non-methadone group, but only shown in the younger children (≤6 years), who also had higher maximum pain scores in the methadone group. All outcomes were similar between analgesia groups in the older children (>6 years). The shift in LOS findings after IPW adjustment in the overall sample was primarily driven by age-related confounding, likely influenced by the unbalanced sample sizes between the methadone (n = 30) and non-methadone (n = 196) groups among younger children. This imbalance may have led to poor covariate overlaps in propensity scores and increased variance in outcome estimates. The observed longer LOS in this subgroup warrants further investigation in larger prospective cohorts.

Caudal morphine combined with a fentanyl bolus is the most commonly utilized analgesic approach for children younger than six years old for cardiac surgery at our institution. Patients older than six historically received either fentanyl or sufentanil infusions. After 2020, sufentanil became less available due to supply chain issues, whereby our institution increasingly relied on methadone for intraoperative analgesia. It is difficult to attribute hospital stays greater than 72 h directly to intraoperative methadone administration, but instead to other patient and surgical factors. This finding underscores the complexity of perioperative care in pediatric cardiac surgery, where multiple confounding factors (i.e., age, sex, race, STAT categories, intraoperative MME/kg, etc.) contribute to postoperative recovery and hospitalization duration.

The pharmacologic profile of methadone includes µ-opioid agonism, N-methyl-d-aspartate (NMDA) receptor antagonism, and inhibition of serotonin and norepinephrine reuptake. Methadone administered via the intravenous route has a fast onset of action, with analgesic effects within 5 min. Pain relief lasts around 4–8 h, similar to morphine, but may last even longer [10,17,18]. Methadone has a significantly longer elimination half-life than morphine, 15–60 h versus 5–6 h, respectively. Opioid tolerance inversely affects the duration of methadone, where opioid-tolerant individuals can expect 24 h duration and opioid-naïve patients can expect 55 h [8,19]. In addition to its analgesic benefits, methadone demonstrates more prolonged analgesia, opioid sparing effects, and the potential to reverse the tolerance to short-acting opioids when compared to other opioids commonly used in surgical settings. There is also evidence that intraoperative use of methadone may mitigate the onset of chronic postoperative pain [8].

While intraoperative methadone provided effective analgesia with a reduced immediate postoperative opioid requirement, its sustained analgesic effects may require additional and repeated postoperative methadone doses, as we found in children undergoing non-cardiac surgeries [10]. This suggests that a tailored, multidose methadone-based patient-specific analgesic regimen may be necessary to optimize pain management throughout hospitalization [6]. The study, therefore, underscores the importance of future evaluation of methadone’s pharmacokinetics for precision dosing and long-term benefits in pediatric cardiac surgical populations.

While the study provides some new knowledge into the analgesic efficacy and safety of intraoperative methadone use in children undergoing cardiac surgery, the following limitations need to be considered. First, this is a large retrospective study design using a single center’s data from electronic health records and the Society of Thoracic Surgeons (STS) Pediatric Cardiac Surgery Database. The retrospective nature of this study may introduce inherent biases, while the single center dataset can limit the generalizability and external validity of the findings. This is the first detailed analysis of the effect of intraoperative methadone compared with non-methadone analgesia in a large cohort of pediatric patients undergoing cardiac surgery. The large, single-centered dataset supports the conclusions without major confounders, including multicenter perioperative practice and patient variability. It is clear, however, that large prospective and multicentered studies are needed to corroborate and expand on these study findings in children. Second, this study was not designed or powered as a formal noninferiority trial; therefore, conclusions about comparable effectiveness are preliminary rather than confirmatory. Further prospective studies with predefined noninferiority margins and larger sample sizes are needed to more rigorously evaluate the clinical comparability of methadone versus non-methadone-based analgesia. Third, we studied intraoperative administration of IV methadone at a dose of 0.1 mg/kg (max 10 mg). Studies in adult cardiac surgical patients utilized higher doses (0.3 mg/kg) of intraoperative methadone [20,21]. A dose finding pharmacokinetic study exploring the ideal dosing regimens in children undergoing cardiac surgery in the setting of cardiopulmonary bypass may provide insights into optimizing perioperative pain management while ensuring patient safety. Fourth, this study did not proactively assess prolongation of QTc in the postoperative period with intraoperative use of methadone. We showed earlier that in children undergoing non-cardiac surgery [10,11] and adults undergoing cardiac surgery [22], perioperative methadone did not prolong QTc interval and/or cause methadone-related ventricular arrhythmias. Large prospective and multicenter studies are needed to demonstrate methadone’s safety and effectiveness in pediatric cardiac surgical patients. Though our study suggests that intraoperative methadone administration seems to provide immediate postoperative analgesia on the day of surgery, optimized intraoperative dosing and subsequent postoperative methadone administration [10] may be needed for sustained analgesia in in-hospital settings.

## 5. Conclusions

In conclusion, this first and large retrospective study in pediatric cardiac surgery patients showed that postoperative opioid use, pain, and antiemetic use were similar among patients who were administered low-dose intraoperative methadone compared to non-methadone intraoperative analgesic options. Since a low dose of intraoperative methadone is highly unlikely to prolong hospital stay significantly, longer hospital stays in younger children warrant further investigation. Given the promising yet nuanced findings of this study, large-scale prospective studies on the perioperative use of methadone in pediatric cardiac patients are warranted. Future research should aim to refine dosing strategies, assess long-term safety outcomes, and determine the most effective multimodal analgesic approaches that incorporate methadone while minimizing opioid-related adverse effects. By doing so, clinicians can enhance pain management strategies and potentially improve postoperative outcomes in children undergoing cardiac surgery.

## Figures and Tables

**Table 1:** Baseline Characteristics by Methadone Analgesia Groups.

Variable	Variable Value	Total n = 287	No Methadone n = 220 (77%)	Methadone n = 67 (23%)	*p*-Value
Age, mean (SD)	years	3.83 (4.87)	2.7 (3.9)	7.6 (5.9)	<0.001
Age Groups, n (%)	≤6 years	226 (78.7%)	196 (89%)	30 (45%)	<0.001
	>6 years	61 (21.3%)	24 (11%)	37 (55%)	
Sex, n (%)	Female	118 (41.1%)	93 (42%)	25 (37%)	0.470
	Male	169 (58.9%)	127 (58%)	42 (63%)	
Race, n (%)	White	207 (72.1%)	157 (71%)	50 (75%)	0.361
	Black	21 (7.3%)	19 (9%)	2 (3%)	
	American Indian	3 (1%)	1 (0%)	2 (3%)	
	Multiple Race	1 (0.3%)	1 (0%)	0 (0%)	
	Other or Asian	2 (0.7%)	2 (1%)	0 (0%)	
	Not Specified	4 (1.4%)	3 (1%)	1 (1%)	
	Patient Declined	49 (17.1%)	37 (17%)	12 (18%)	
Surgery Type, n (%)	CPB Cardiovascular	271 (94.4%)	210 (95%)	61 (91%)	0.020
	No CPB Cardiovascular	12 (4.2%)	9 (4%)	3 (4%)	
	Other	1 (0.3%)	1 (0%)	0 (0%)	
	VAD Operation Done With CPB	3 (1%)	0 (0%)	3 (4%)	
STAT category, n (%)	1	164 (58%)	132 (60%)	32 (50%)	0.331
	2	93 (32.9%)	69 (32%)	24 (38%)	
	3	21 (7.4%)	15 (7%)	6 (9%)	
	4	5 (1.8%)	3 (1%)	2 (3%)	
Weight, mean (SD)	kg	18.25 (18.53)	14.5 (14.8)	30.6 (23.7)	<0.001

**Table 2:** Intraoperative Medications by Methadone Analgesia Groups.

Variable	Total n = 287	No Methadone n = 220 (77%)	Methadone n = 67 (23%)	*p*-Value
Caudal, n (%)	173 (60.3%)	171 (78%)	2 (3%)	<0.001
Fentanyl, n (%)	276 (96.2%)	220 (100%)	56 (84%)	<0.001
Methadone, n (%)	67 (23.3%)	0 (0%)	67 (100%)	<0.001
Sufentanil, n (%)	35 (12.2%)	34 (15%)	1 (1%)	0.001
Hydromorphone, n (%)	1 (0.3%)	1 (0%)	0 (0%)	0.999
Ketamine, n (%)	1 (0.3%)	0 (0%)	1 (1%)	0.233
Intraoperative Opioid MME/kg, Median (IQR)	0.5 (0.39–0.56)	0.5 (0.4–0.6)	0.5 (0.4–0.5)	0.649

**Table 3:** Unadjusted associations between methadone groups and outcomes.

Outcome Variable	Total n = 287	No Methadone n = 220 (77%)	Methadone n = 67 (23%)	*p*-Value
**Postoperative Opioid Use in MME/kg, median (IQR)**				
POD 0	0.43 (0.16–1.03)	0.5 (0.2–1.1)	0.3 (0.1–0.7)	0.005
POD 1	0.1 (0.05–0.19)	0.1 (0.1–0.2)	0.1 (0–0.2)	0.718
POD 2	0.07 (0.04–0.15)	0.1 (0–0.1)	0.1 (0–0.2)	0.052
Total (POD 0–4)	0.69 (0.3–1.31)	0.7 (0.3–1.4)	0.5 (0.3–1.1)	0.132
**Average Pain Score, mean (SD)**				
POD 0	2.05 (1.49)	2.1 (1.5)	1.9 (1.3)	0.422
POD 1	1 (1.01)	1 (1)	1 (1.1)	0.890
POD 2	1.02 (1.46)	1 (1.4)	0.9 (1.5)	0.680
**Max Pain Score, mean (SD)**				
POD 0	7.54 (2.67)	7.6 (2.7)	7.3 (2.7)	0.403
POD 1	4.54 (3.55)	4.6 (3.6)	4.4 (3.4)	0.718
POD 2	3.92 (3.37)	3.9 (3.3)	4.1 (3.6)	0.739
**Any Postoperative Antiemetic, n (%)**	59 (20.7%)	32 (15%)	27 (40%)	<0.001
POD 0	43 (15%)	22 (10%)	21 (31%)	<0.001
POD 1	24 (8.4%)	11 (5%)	13 (19%)	<0.001
POD 2	16 (5.6%)	7 (3%)	9 (13%)	<0.001
**Postoperative Hospital LOS, median (IQR)**	5 (3–8)	5 (3–8)	5 (4–9.8)	0.089
**ICU LOS, median (IQR)**	2.1 (1.15–3.21)	2.1 (1.2–3.2)	2.1 (1.2–4)	0.823
**Reintubated, n (%)**	30 (10.5%)	21 (10%)	9 (13%)	0.366
**Naloxone, n (%)**	6 (2.1%)	5 (2%)	1 (1%)	0.999

Abbreviations: POD, post-operative day; kg, kilogram; mg, milligrams; MME, morphine milligram equivalents; LOS = length of stay.

**Table 4:** IPW adjusted associations between methadone groups and outcomes.

Outcome Variable	Total n = 567.6	No Methadone n = 281.9 (49.7%)	Methadone n = 285.68 (50.3%)	*p*-Value
**Postoperative Opioid Use in MME/kg, median (IQR)**				
POD 0	0.5 (0.2–1.1)	0.4 (0.2–1)	0.6 (0.2–1.2)	0.496
POD 1	0.1 (0.1–0.2)	0.1 (0.1–0.2)	0.1 (0.1–0.3)	0.311
POD 2	0.1 (0–0.2)	0.1 (0–0.1)	0.1 (0.1–0.4)	0.017
Total (POD 0–4)	0.7 (0.3–1.4)	0.7 (0.3–1.2)	0.7 (0.4–1.8)	0.139
**Average Pain Score, mean (SD)**				
POD 0	1.99 (1.38)	2.00 (1.54)	1.97 (1.19)	0.889
POD 1	1.02 (1.04)	0.94 (0.99)	1.12 (1.11)	0.450
POD 2	1.08 (1.58)	1.01 (1.42)	1.15 (1.74)	0.719
**Max Pain Score, mean (SD)**				
POD 0	7.60 (2.74)	7.23 (2.95)	8.03 (2.43)	0.082
POD 1	4.76 (3.65)	4.30 (3.62)	5.27 (3.66)	0.236
POD 2	4.44 (3.43)	3.74 (3.33)	5.15 (3.45)	0.068
**Any Postoperative Antiemetic, n (%)**	108.6 (19.2)	49.1 (17.5)	59.5 (20.8)	0.557
POD 0	85.1 (15.0)	35.7 (12.7)	49.4 (17.3)	0.359
POD 1	41.0 (7.2)	17.7 (6.3)	23.3 (8.1)	0.565
POD 2	31.2 (5.5)	11.2 (4.0)	19.9 (7.0)	0.356
**Postoperative Hospital LOS, median (IQR)**	5 (3–10)	4 (3–8)	8 (4–23.4)	0.002
**ICU LOS, median (IQR)**	2.2 (1.2–4.9)	2.1 (1.1–3.1)	2.8 (1.8–6.3)	0.018
**Reintubated, n (%)**	65.5 (11.5)	27.4 (9.7)	38.1 (13.3)	0.515
**Naloxone, n (%)**	10.0 (1.8)	8.0 (2.9)	2.0 (0.7)	0.171

Abbreviations: IPW, inverse probability weighted; POD, post-operative day; kg, kilogram; mg, milligrams; MME, morphine milligram equivalents; LOS = length of stay.

**Table 5:** IPW adjusted associations between methadone group vs non-methadone group (reference) and clinical outcomes over postoperative days 0–2 and stratified by age groups.

Outcomes	Opioid Use in MME/kg	Average Pain Score	Max Pain Score	Postoperative Antiemetic	Postoperative Hospital LOS
**Estimates**	Mean Difference 95% CI	Mean Difference 95% CI	Mean Difference 95% CI	Relative Risk 95% CI	Mean Ratio 95% CI
**Overall (n = 287)**	*p* = 0.73 −0.03 (−0.22, 0.16)	*p* = 0.66 −0.07 (−0.40, 0.26)	*p* = 0.99 −0.002 (−0.76, 0.76)	*p* = 0.56 1.19 (0.67, 2.1)	*p* < 0.001 2.62 (1.55, 4.41)
**Age ≤ 6 years** **(n = 226, 79%)**	*p* = 0.20 0.17 (−0.09, 0.44)	*p* = 0.87 0.03 (−0.37, 0.44)	*p* = 0.02 0.91 (0.15, 1.68)	*p* = 0.8 1.14 (0.41, 3.23)	*p* < 0.001 2.14 (1.3, 3.55)
**Age > 6 years** **(n = 61, 21%)**	*p* = 0.76 0.02 (−0.22, 0.27)	*p* = 0.32 0.54 (−0.55, 1.64)	*p* = 0.68 0.4 (−1.59, 2.4)	*p* = 0.19 1.86 (0.73, 4.72)	*p* = 0.36 1.13 (0.87, 1.46)

Abbreviations: kg, kilogram; mg, milligrams; MME, morphine milligram equivalents; LOS, length of stay.

## Data Availability

UPMC Department of Anesthesiology and Perioperative Medicine, University of Pittsburgh archived dataset analyzed.

## Supplementary Materials

The following supporting information can be downloaded at: https://www.mdpi.com/article/10.3390/children12050567/s1, Table S1: Propensity Score Covariate Balancing.

## References

[1] Stanley T.H. (2014). The fentanyl story. J. Pain.

[2] Pathan H., Williams J. (2012). Basic opioid pharmacology: An update. Br. J. Pain.

[3] Scholz J., Steinfath M., Schulz M. (1996). Clinical pharmacokinetics of alfentanil, fentanyl and sufentanil. An update. Clin. Pharmacokinet..

[4] Anand K.J., Willson D.F., Berger J., Harrison R., Meert K.L., Zimmerman J., Carcillo J., Newth C.J., Prodhan P., Dean J.M. (2010). Tolerance and withdrawal from prolonged opioid use in critically ill children. Pediatrics.

[5] Nguyen K.N., Byrd H.S., Tan J.M. (2016). Caudal analgesia and cardiothoracic surgery: A look at postoperative pain scores in a pediatric population. Paediatr. Anaesth..

[6] Rosen K.R., Rosen D.A. (1989). Caudal epidural morphine for control of pain following open heart surgery in children. Anesthesiology.

[7] Horlocker T.T., Vandermeuelen E., Kopp S.L., Gogarten W., Leffert L.R., Benzon H.T. (2018). Regional Anesthesia in the Patient Receiving Antithrombotic or Thrombolytic Therapy: American Society of Regional Anesthesia and Pain Medicine Evidence-Based Guidelines (Fourth Edition). Reg. Anesth. Pain Med..

[8] Ramaiah V.K., Kharasch E.D. (2024). Methadone and Enhanced Recovery After Surgery: Concepts and Protocols. Anesth. Analg..

[9] Garcia S., Mali M., Grewal A. (2024). Pro: Methadone Should Be Used as a Part of Enhanced Recovery After Cardiac Surgery Protocol. J. Cardiothorac. Vasc. Anesth..

[10] Sadhasivam S., Aruldhas B.W., Packiasabapathy S., Overholser B.R., Zhang P., Zang Y., Renschler J.S., Fitzgerald R.E., Quinney S.K. (2021). A Novel Perioperative Multidose Methadone-Based Multimodal Analgesic Strategy in Children Achieved Safe and Low Analgesic Blood Methadone Levels Enabling Opioid-Sparing Sustained Analgesia With Minimal Adverse Effects. Anesth. Analg..

[11] Ye J., Myung K., Packiasabapathy S., Yu J.S., Jacobson J.E., Whittaker S.C., Castelluccio P., Drayton Jackson M., Sadhasivam S. (2020). Methadone-Based Multimodal Analgesia Provides the Best-in-Class Acute Surgical Pain Control and Functional Outcomes with Lower Opioid Use Following Major Posterior Fusion Surgery in Adolescents with Idiopathic Scoliosis. Pediatr. Qual. Saf..

[12] Barnett A.M., Machovec K.A., Ames W.A., Homi H.M., Turi J.L., Koo J., Fuller M., Jooste E.H. (2020). The effect of intraoperative methadone during pediatric cardiac surgery on postoperative opioid requirements. Paediatr. Anaesth..

[13] Santa Cruz Mercado L.A., Liu R., Bharadwaj K.M., Johnson J.J., Gutierrez R., Das P., Balanza G., Deng H., Pandit A., Stone T.A.D. (2023). Association of Intraoperative Opioid Administration With Postoperative Pain and Opioid Use. JAMA Surg..

[14] Nielsen S., Degenhardt L., Hoban B., Gisev N. (2016). A synthesis of oral morphine equivalents (OME) for opioid utilisation studies. Pharmacoepidemiol. Drug Saf..

[15] Merkel S.I., Voepel-Lewis T., Shayevitz J.R., Malviya S. (1997). The FLACC: A behavioral scale for scoring postoperative pain in young children. Pediatr. Nurs..

[16] Krechel S.W., Bildner J. (1995). CRIES: A new neonatal postoperative pain measurement score. Initial testing of validity and reliability. Paediatr. Anaesth..

[17] Aruldhas B.W., Quinney S.K., Overholser B.R., Heathman M.A., Masters A.R., Ly R.C., Gao H., Packiasabapathy S., Sadhasivam S. (2021). Pharmacokinetic modeling of R and S-Methadone and their metabolites to study the effects of various covariates in post-operative children. CPT Pharmacomet. Syst. Pharmacol..

[18] Packiasabapathy S., Aruldhas B.W., Zhang P., Overholser B.R., Quinney S.K., Sadhasivam S. (2021). Novel associations between CYP2B6 polymorphisms, perioperative methadone metabolism and clinical outcomes in children. Pharmacogenomics.

[19] D’Souza R.S., Esfahani K., Dunn L.K. (2023). Pro-Con Debate: Role of Methadone in Enhanced Recovery After Surgery Protocols-Superior Analgesic or Harmful Drug?. Anesth. Analg..

[20] Murphy G.S., Avram M.J., Greenberg S.B., Shear T.D., Deshur M.A., Dickerson D., Bilimoria S., Benson J., Maher C.E., Trenk G.J. (2020). Postoperative Pain and Analgesic Requirements in the First Year after Intraoperative Methadone for Complex Spine and Cardiac Surgery. Anesthesiology.

[21] Murphy G.S., Szokol J.W., Avram M.J., Greenberg S.B., Shear T.D., Deshur M.A., Vender J.S., Benson J., Newmark R.L. (2017). Clinical Effectiveness and Safety of Intraoperative Methadone in Patients Undergoing Posterior Spinal Fusion Surgery: A Randomized, Double-blinded, Controlled Trial. Anesthesiology.

[22] McClain M.R., Subramaniam K., Cheema R., Lavage D.R., Lin H.S., Sultan I., Sadhasivam S., Howard-Quijano K. (2025). Intraoperative Methadone in Adult Cardiac Surgical Patients and Risks for Postoperative QTc Prolongation. J. Cardiothorac. Vasc. Anesth..

