# Supplementary material for: Intraoperative Methadone Versus Non-Methadone Analgesia in Pediatric Cardiac Surgery: A Retrospective Cohort Study
Source: Children (Basel). 2025 Apr 28;12(5):567. doi: 10.3390/children12050567 (PMC12109820; doi:10.3390/children12050567)
Supplement: Supplementary file 1 [file children-12-00567-s001.zip › children-3541212-supplementary.pdf]

Supplemental Table 1. Propensity Score Covariate Balancing		
**Propensity Score Model Predictor**	**Unweighted SMD**	**IPW SMD**
Age	0.981	0.056
Sex	0.160	0.106
Race (BIPOC Vs Not BIPOC)	0.084	0.091
STAT Category	0.224	0.027
Intraoperative MME / kg	0.093	0.074

Displaying between treatment group standardized mean differences (SMD) for each predictor used in the propensity score modeling. We chose SMD <= 0.2 as our cutoff for balanced covariates. All covariates showed SMDs below our threshold post weighting.

Abbreviations: kg, kilogram; MME, morphine milligram equivalents, BIPOC, Black, indigenous or people of color; SMD, standardized mean differences; IPW, inverse probability weighting
